# A Few Dental Points

**Published:** 1898-10

**Authors:** F. Mackinzie


ARTICLE VII.

### A FEW DENTAL POINTS.

DR. F. MACKINZIE,

It is often difficult to remove the impression of some cases. Ask the patient to cough, and this will raise the soft palate and the air will be admitted between the composition and the palate. The impression may then be readily removed. The nauseous effects of the composition may generally be overcome by bringing the patient's head well forward after the impression has been got into position. To remove an impression in a lower case, insert the index fingers one on each side and press the cheeks outward from within.

The rattle of a full upper and lower case may be overcome somewhat by this method. Build up the case in the usual way in wax, but in packing remove the four end molars and pack white rubber in instead of the teeth. Thus, there will be four pads of softer material than the teeth, which, when they come in contact, will reduce the rattle.

When packing gum block cases, it is better to pack them as soon as possible after having boiled out the wax.

The plaster seems to undergo some change, which allows the points to open when the flasks are well heated. There are various ways of preventing the rubber from getting between the joints; one of the best is to get the joints fitted so that the whole of the side of the one block comes in contact with the whole side of the other. If only the labial edges of the blocks are in contact, a wedge-shaped space is left behind, through which the rubber often contrives to run.

When the case is built up in wax with all the joints fitted, each ought to be taken off in turn, and all traces of wax and dial removed from the joints. Then wax on again, but leave spaces opposite each joint, so that thin plaster; or osteo, or whatever is used, may be placed in the joint. The object is to keep the wax out of the joint entirely; finally wax up and put the case in the flask.

When removing teeth from an old vulcanite case, put a little wax on when heating in the gas. This will soften the vulcanite and render removal of the teeth easy. Another method is to boil the plate in water until soft; this will do away with the unpleasant smells with which we are all familiar.

When zinc and lead have become mixed, make a cone shaped mold in the sand and pour in the melted metal. When the zinc has set, lift out of the sand, and the lead, still melted, will remain at the bottom; or leave the whole mass to set, and cut off the lead afterward.

The following method is useful for gold filling in an incisor, in which the corner of the tooth is broken off, and it is impossible to get an opposing point. Prepare the tooth for filling in the ordinary way, either for wedging, or with retaining pits, then on the lingual wall cut out a small key or dove-tail, taking care that it is in the strongest part, not too near the cutting edge of the tooth, or too near the base of the cavity, which in either case would leave a weak part in the tooth. The part where the opposing point ought to be need not be taken into consideration in preparing the cavity. Then proceed to fill the base of the cavity in the usual way, and build the gold up to and into the key, finish and polish. The filling will be quite as firm and perfect as if it were filled by means of an opposing pit. The tooth ought to be strong enough to bear the cutting out of the key, otherwise it will soon come to grief, but in suitable cases the tooth and filling ought to last a long time.—*British Journal.*

